# Peer review: My article was rejected by the journal I edit

**DOI:** 10.36834/cmej.70700

**Published:** 2020-08-06

**Authors:** Marcel D’Eon

**Affiliations:** 1 Medical College of Georgia, Augusta University, Augusta, USA; 2 Professor Emeritus, University of Saskatchewan, Saskatchewan, Canada

My article was rejected by the journal I edit: peer review is alive and well at the CMEJ. I am writing this editorial about peer review propelled in large part because a paper I wrote with a team of colleagues not connected with the journal was carefully reviewed, thoughtfully considered, and then declined by the associate editor. This is a testament not just to the robust and rigorous peer review process valued and followed at the CMEJ but to the people that make the journal work, the people who make the processes and policies function properly. While this decision stung (just as we acknowledge in our communication to authors that every decline decision is disappointing), I want to praise the people and the processes, the heart of our peer review, for skillfully declining to publish my paper as submitted.

Arguably, peer review is at the heart of what makes an academic journal credible, if not great. This is not to claim that we are unique or better than our sister journals but only to state that we must be doing something right at least some of the time, which it seems, is the best that can be said of peer review in general.^1,2^

As with all decision-making processes there are inevitable errors even in careful peer review. It seems that academic peer review is no different or maybe a bit worse than other similar processes. We reject papers that will eventually (when published somewhere else) contribute to the literature and help move our knowledge of the field along, sometimes in dramatic ways. These are false negatives. We reject papers when we should not have rejected them. Others are accepted and published in our journals but no one else ever reads them or they turn out to be false and/or fabricated. These are the false positives, the papers accepted that should have been declined. What does this look like in practice?

I can think of some examples from my own experience. Years ago, I found in a leading journal a paper on problem-based learning that had numerous serious and glaring weaknesses that left me bewildered. I found it hard to comprehend how such a paper could possibly get past the peer review process and end up published. Embarrassingly, I sometimes wonder how a few of my early papers ever were accepted. On the other hand, a paper I wrote with a colleague that introduced and explained an innovative (even radical) approach to the treatment of aggregated self-assessments that I helped develop was rejected by a top journal because the reviewers and editors could only think about the topic in the conventional way. Try as they did (I assume they tried), they could not see things differently. There are also numerous instances where thoughtful critique helped shape my ideas so that I could refine them and present them more convincingly on the way to being published. There’s good and not so good in this business.

Siler and team found (maybe shockingly), that rejected manuscripts that were eventually published in prestigious journals were more cited than manuscripts accepted first time.^2^ Furthermore, they found that three of the top medical journals rejected the top 14 cited articles. Peer review is not always very good at identifying and publishing the top articles, or at recognizing and promoting excellence and innovation. This finding is distressing for those of us hoping to move the theory and practice of medical education forward.

Where peer review seems to be more effective is identifying and declining those articles that are not likely to contribute to the field.^2^ Peer review can sift through the hundreds and thousands of submissions and correctly identify the vast majority of those that are low in quality and potential. While it is not the most attractive or compelling strength of the peer review process - finding and discarding the space junk does not have the same appeal as discovering and refining the gems and treasures - it does provide a valuable service to the community. Furthermore, if we worked hard to accept all those top studies and articles (reduced the number of false negatives) we could easily end up publishing more of the less desirable articles (increase the number of false positives). Pick your poison.

Recently, with the rush to find treatments and a vaccine for the COVID-19 virus, journals are fast tracking articles and publishing them at astonishing speeds.^3^ This is a very good practice as it makes the results of research publicly available as scientists try to build on the work of others as a short cut to finding answers to pressing needs worldwide. Predictably, there have been some mistakes. Some papers have been withdrawn and others soundly criticized by prominent experts in the field. What happened in the peer review process in those cases of false positives? Likely, in the rush to expedite the vanquishing of the COVID-19 virus that so far has held the entire world captive, the editors took chances that they would publish some false positives. This is a good thing, but in these circumstances consumers of research need to be even more vigilant to critique the peer reviewed, published literature (as I did with that article on PBL). The emergency approval and subsequent withdrawal of hydroxychloroquine by the FDA was a similar phenomenon at play, of rushing to find a break-through discovery.

Let’s entertain the notion that we can make peer review better, not perfect, but just better. Here are some potential avenues for improvement

1. Journals can create and implement better and easier processes to facilitate the work of the reviewers. At the CMEJ we have undertaken a study of the review process by first asking reviewers about their experiences and then their ideas for making the process better for them and the authors.
2. Journals can treat reviewers as valued colleagues and not just lowly cogs in the publication mills of academia. Many if not most journals print the names of those who have reviewed for them in the last year; some provide letters that can be used on CVs; there are formal ways to provide reviewers with academic credit for completing reviews; at the CMEJ we thank our reviewers each time they complete a review and many of the editors add a personal note to the automatically generated email test; and I send our top reviewers (about 50 of them) a short somewhat personalized “Thank you” email directly from my own email account.
3. We can better train our reviewers.^4^ Graduate programs can include specific skills of critical appraisal along with supervised “team” reviews of submissions. At the CMEJ we encourage these forms of peer review and we find we receive very high quality reviews from those collaborations (though we have no research data to support our claim). Journals can also make the directions easy to follow and can even provide some basic on-line, self-directed training for those just starting out or wanting a refresher.
4. We can find and keep more reviewers. Marketers know that it is easier (and better) to keep your existing customers than to find new ones. Several of the suggestions I have described above will help with retention and recruitment. Having more reviewers means that everyone does not have as much work to do as it can be spread out over many willing hands which might make this volunteer job a little more attractive.
5. Authors can create an informal or formal internal peer review process and/or engage the services of an editor. This will very likely improve the paper and make it more likely to pass the editorial desk review and go to peer review. Many papers are declined not because the ideas are weak or the methodology inappropriate, but because the quality of the writing is poor and the editors, understandably, do not want to invest the hours needed to work with the authors to bring that aspect of the paper up to standard. Sometimes papers are rejected because the valuable parts are unrecognizable.
6. Finally, journal editors can take more time and invest more effort when making these important decisions. In some journals, editors consider together the reviews and their own critiques of the papers and make collaborative decisions.^4^ The thoughtful deliberations may yield better decisions.

Generally, peer review does a reasonable job of what we expect from it, but we can do better. It is certainly better than no peer review or other systems or non-systems that we know about. I will conclude, then, borrowing heavily from the title of Aaron Carroll’s New York Times article from 2018:^4^ Peer review is the worst way to judge research except for all the other ways. On that note, let me introduce the papers that have persevered through the rigours of our peer review process and are featured in this issue of the CMEJ. Finally, let me thank once again all the reviewers who gave of their time and talent to help bring these and all our articles to our readers.

MacLean and her team in “A pilot study of a longitudinal mindfulness curriculum in undergraduate medical education” described a pilot mindfulness curriculum and the results of their three-year study. They found that mindfulness scores correlated positively with those of empathy and resilience and negatively with perceived stress. They recommended further study.

Fung and team, in “Learning by chance: Investigating gaps in transgender care education amongst family medicine, endocrinology, psychiatry and urology residents” interviewed residents from four different specialties likely to provide care to transgender people. They explored resident training experiences related to caring for trans people. They found that there was a lack of education around trans care. They believe medical education needs to address the healthcare disparities of this population.

In the article “Medical students’ personal experiences, religion, and spirituality explain their (dis)comfort with a patient’s religious needs,” Schmidt and team studied how students’ own religious and spiritual background reflected their comfort level when addressing a patient with religious needs. They found that students with a personal religious or spiritual background had a higher level of comfort with discussing a patients’ religious concerns. They hope their study will aid medical educators in teaching mind-body-spirit care.

Sanaee and team in their article “Practical solutions for implementation of *transition to practice* curricula in a competency-based medical education model,” constructed a definition and developed initial curriculum content for *Transition to Practice* (TTP). They want the results of their study to provide a prototype for curriculum development and delivery within the competency-based medical education model.

Acai and co-authors in “The role of gender in the decision to pursue a surgical career: A qualitative, interview-based study,” conducted interviews in order to recognize the role of gender in medical students’ decision to pursue a surgical career. They found that gender is more likely to be a barrier than a motivator.

Beavers et. al, in their article “Perceptions of preparedness: How hospital-based orientation can enhance the transition from academic to clinical learning,” explored medical students’ perceptions of hospital-based orientation as a useful tool for transitioning from academic to clinical learning. They argued that the orientation played an important role in learners’ preparedness at the *unit/service* and *individual* levels.

Dawe and McKelvie in “International health experiences in postgraduate medical education: A meta-analysis of their effect on graduates’ clinical practice among underserved populations” completed a systematic review to evaluate the effect of postgraduate international health experiences (IHE) participation on the future careers of clinicians in their work with domestic and/or international underserved populations. They found that participation in an IHE may cause an increase in care for underserved populations, though they recommended further research in this area.

In “The Women in Medicine Summit (WiMS): Engaging students to identify and address gender-associated challenges in medicine” Jung and co-authors described a new student-led medical conference (WiMS) that was aimed at discussing gender-distinctive challenges in medicine. They reported positive feedback from the WiMs attendees. They concluded that other medical schools could benefit from similar curricular initiatives.

“CBME framework to promote transition to senior” by Acker and team described their CBME framework that combines the traditional method of transitioning residents to senior level independent overnight call with a workplace-based assessment strategy. They found that their method increased confidence and improved comfort for transitioning and transitioned residents. They want their framework to be expanded to other departments

In “Ice Cream Rounds: The implementation of peer support debriefing sessions at a Canadian medical school” Hiranandani and Calder-Sprackman described the implementation of Ice Cream Rounds (ICRs) to improve wellness by providing students a safe environment to discuss challenging clinical and professional experiences. They reported that all respondents would recommend ICRs to other medical students.

Zhao and D’Eon, in “Five ways to get a grip on grouped self-assessments of competence for program evaluation,” offered ideas for the use of grouped self-assessments to prevent future misuse and to enhance program evaluations. They hope that researchers will review their use of grouped self-assessments to improve practices.

In the article, “Reclaiming physician identity: It’s time to integrate ‘Doctor as Person’ into the CanMEDS framework,” Dagnone and team proposed formalizing ‘Doctor as Person’ as the eighth role in the CanMEDS framework. They proposed that this role would aid in increasing the humanity in medical care.

In “Creating space for Indigenous healing practices in patient care plans,” Poudel and Chaudhary wrote a letter affirming Logan and team’s previously published article. They applauded Logan’s ideas for integration of indigenous healing practices into the practice of modern medicine; and suggested that the model of study can be replicated in larger geographical sections.

Brocklebank and Jowsey have contributed an image to the CMEJ: “Interprofessional education or silo education?” They depicted the difficulties that practitioners may have in working in interprofessional teams having trained in separate buildings and without having interacted much at all.

Finally, Nancy Duan in “The universe and I: An exploration of the self and our place in the world” shared a three-part image. She used her images to explore therelationship of the individual to the world around them.

Enjoy!


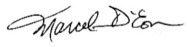


Marcel D’Eon, MEd, PhD Editor, CMEJ
